# Admission factors associated with ICU readmission in onco-hematological critically ill patients: a retrospective cohort study

**DOI:** 10.1186/cc14669

**Published:** 2015-09-28

**Authors:** Leandro U Taniguchi, Cinthia M Rodrigues, Ellen MC Pires, Jorge PO Feliciano, Jose M Vieira

**Affiliations:** 1 Education and Research Institute, Hospital Sírio-Libanês, São Paulo, São Paulo, Brazil

## Introduction

Despite initial recovery, many onco-hematological patients require ICU readmission, which is a burdensome condition with implications both for mortality and quality of life. Nevertheless, little has been published regarding identification of risk factors for ICU readmission in the onco-hematological population of critically ill patients.

## Objective

To determine the admission factors associated with ICU readmission among onco-hematological patients.

## Methods

Retrospective cohort study using an ICU database (Sistema Epimed™) from a tertiary oncological center. From January 2012 to December 2012, 2629 patients were admitted to our ICU. Forty-nine percent (1155 patients) had onco-hematological conditions and were the subject of this retrospective study. We used univariate and multivariate analysis to identify at admission risk factors associated with later ICU readmission in the same hospitalization period.

## Results

One hundred and five patients (9.1%) were readmitted after ICU discharge. Patients readmitted were sicker compared with the nonreadmitted group (SAPS III of 49 (IQR 33-53) vs. 37 (28-49) respectively, *p *<0.001), had a nonsurgical reason for hospitalization (79% vs. 40% respectively, *p *<0.001), more frequently came from the ward (36.2% vs. 13.9% respectively, *p *<0.001), had longer hospital length of stay (LOS) before ICU admission (15 days (8.5-29) vs. 1 day (0-2) respectively, *p *<0.001), more frequently came to the ICU due to respiratory failure (19.2% vs. 4.7% respectively, *p *<0.001) or neurological disturbances (24% vs. 8% respectively, *p *<0.001), more frequently required mechanical ventilation (34.6% vs. 18.4% respectively, *p *<0.001) and vasoactive drugs (47.1% vs. 33.3% respectively, *p *<0.001), and had higher hospital mortality (28.6% vs. 13.9% respectively, *p *<0.001). On multivariate analysis, longer hospital LOS before ICU transfer, neurological disturbances and nonsurgical reason for admission were independent risk factors for ICU readmission.

## Conclusion

We identified some risk factors associated with ICU readmission in onco-hematological patients, but unfortunately all are not amenable to interventions. Identification of risk factors at ICU discharge might be more promising.

